# Better digital health data should be the foundation to transform outpatient consultations for people living with long-term conditions

**DOI:** 10.1177/01410768221089020

**Published:** 2022-04-01

**Authors:** Julie Gandrup, Karen Staniland, Charlotte A Sharp, William G Dixon

**Affiliations:** 1 Centre for Epidemiology Versus Arthritis, Division of Musculoskeletal and Dermatological Sciences, School of Biological Sciences, University of Manchester, Manchester, M13 9PL, UK; 2 Rheumatology, Salford Care Organisation Northern Care Alliance, Salford, M6 8HD, UK; 3 The Centre for Primary Care and Health Services Research, The University of Manchester, Manchester, M13 9PL, UK; 4 NIHR Greater Manchester Biomedical Research Centre, Manchester Academic Health Science Centre, University of Manchester, Manchester, M13 9PL, UK

Outpatient departments support disease management for people living with long-term conditions (LTCs) like cardiovascular disease, diabetes and arthritis. The demand for outpatient care is increasing: around one in four people in the UK now live with one or more LTCs,^1^ with outpatient appointment numbers increasing by more than 50% in the decade to 2018–19.^2^

The pandemic led to a rapid increase in the use of technology for consultations. In July 2020, remote consultations accounted for >70% of interactions in primary care, up from 25% the previous year.^3^ This digital transformation is heralded as an opportunity for future care, acknowledging benefits such as reducing travel, reducing the spread of infections and reducing non-attendance. Nonetheless, these opportunities come balanced by challenges. Successful outpatient care requires a clear understanding of how patients’ symptoms and management evolve through time. Through the pandemic, the reduction in good-quality information to inform shared decision making became apparent: virtual care misses the richness of face-to-face consultations, and removes the ability to perform physical examinations.

The National Health Service (NHS) now seeks to ‘build back better’, expanding on its pre-existing vision,^4^ informed by the rapid changes forced by the pandemic.^5^ Outpatient clinics are unlikely to revert to the same pre-pandemic operating model, not least because accelerated digital transformation has delivered many of the above benefits. During this period of change, it is vital that we think carefully about how digitisation can support the collection, collation and presentation of clinical data for excellent care, as well as for other secondary uses.^6^ A strong data foundation for outpatients is particularly important if we are to offset some of the challenges of fewer face-to-face consultations.

This article considers the purpose of a consultation, then explores opportunities for advancing the collection and use of digital health data to transform outpatients. It considers how such data might also be used for other purposes such as planning and research. The article focuses on the collection and presentation of data from structured data entry into the electronic health record (EHR) and the use of integrated electronic patient-generated health data to improve shared decision making^7^ and provide more patient-centred care.

## The model for outpatient consultations

**Figure 1.**
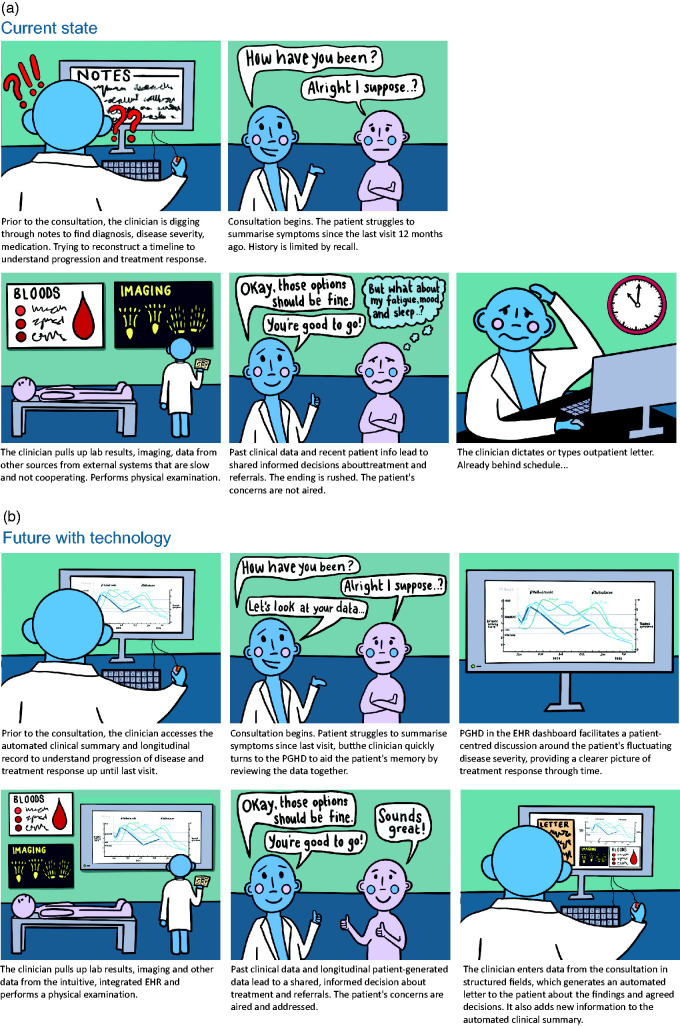
Current and future outpatient consultations. (a) Example of pitfalls in the current outpatient process due to data gaps, illustrated through the story of a 20-min rheumatology consultation. (b) Examples of opportunities from structured, integrated digital health data collected by both patients and clinicians, illustrated through the same 20-min rheumatology consultation. EHR: electronic health record; PGHD: patient-generated health data.

Outpatient visits follow a model unchanged for centuries.^8^ Prior to the consultation, a clinician reviews the referral letter or past visit notes. The consultation proceeds with history taking, examination and investigations, a sequence repeated at follow-up appointments. The clinician’s goals are to gather sufficient information to reach a differential diagnosis and assess disease severity and treatment response to guide management. Patients hope that consultations allow them to explain their concerns, so clinicians can guide them towards better health and wellbeing.^9^ Unfortunately, steps in this process can be imperfect, especially when time is constrained. Efficient elicitation and collation of pertinent information is often challenging, with knock-on consequences for the rest of the consultation (Figure 1(a)). We propose that technology can help improve this.

## Unlocking the potential of electronic health records and structured data capture

Piecing together information by scanning scores of outpatient letters can be time-consuming and frustrating. This is further complicated if records are inaccurate, incomplete or inaccessible. Paper records (still used in around one in four hospital Trusts^10^) may be filed in the wrong order, fallen out or otherwise missing. Digital health records provide easier access but are still a series of free-text documents requiring time to review manually.

While the primary purpose of health data is to guide individual care, such data are also used to understand the healthcare system through planning and research. The lack of structured and coded outpatient data means, amazingly, there is no detailed overview of outpatient services in the UK. For example, we do not know about outpatient case mix or prescribing because diagnosis and medication data are locked away within unstructured letters. National clinical audits mostly rely on manual data entry into an online audit database. These audits provide important insights about which service improvements are required and the impact of interventions, but data collection is highly inefficient and often incomplete.

Structured data collection using EHRs seems an obvious solution for direct care and secondary uses (Figure 1(b)). But the challenges are significant. Clinicians may be reluctant to enter coded information – understandably, investing time to enter data will not be acceptable without direct benefits. Hospital EHR departments equally do not have the capacity to develop multiple bespoke data entry systems for the varied outpatient-based specialties.

**Figure 2.**
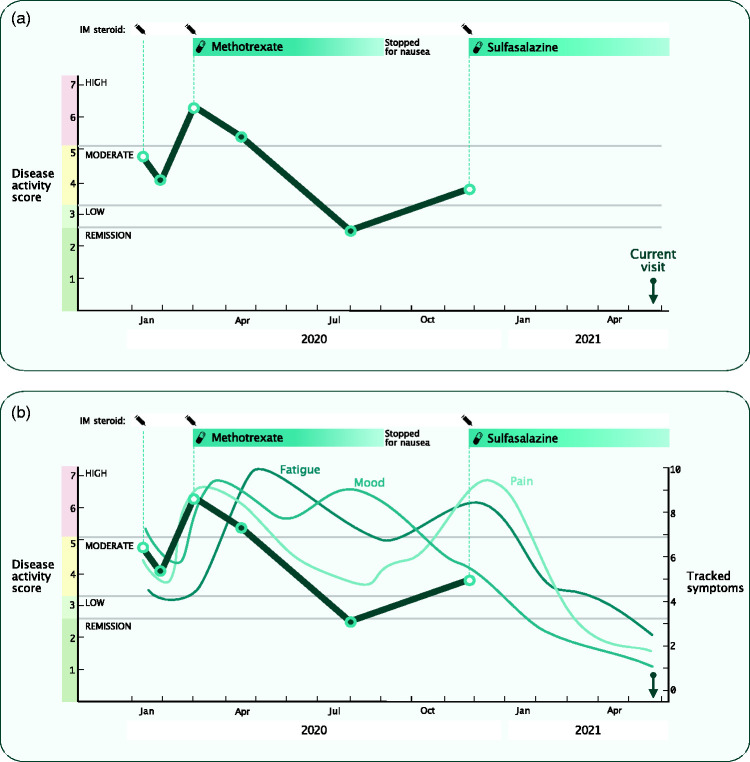
(a) Longitudinal visual record of a hypothetical patient with rheumatoid arthritis. The visualisation starts with a presentation in January 2020 with moderate disease severity. Following initial treatment with intramuscular (IM) steroid, there was initial improvement but then there was a recurrence with worsening disease severity. Methotrexate was commenced at the end of February, after which disease activity improved. Treatment was discontinued in August due to intolerable nausea. The most recent visit found moderate disease activity and the clinician commenced a second disease-modifying anti-rheumatic drug, sulfasalazine, alongside administration of IM steroid injection. The vertical arrow indicates the visit that the patient is currently coming to clinic for. (b) The same longitudinal visual record with real-time patient-generated health data (here symptom tracking of fatigue, mood and pain) added, illustrating good patient benefit from the new treatment.

Could there be a common solution across outpatient-based specialties? It is theoretically possible to design a generic outpatient data collection system for all departments caring for patients with LTCs. All share a need to collect the same core information: demographics, environmental exposures, vital signs, diagnoses, results, medications and – for each disease – the disease-specific outcome measures. By focussing efforts on a common system, data can be standardised using accepted coding terminologies (e.g. Snomed for diagnoses and d + md for medications). Data quality would increase, further supporting interoperability and enabling national statistics. More importantly, it could support better, safer care by reducing inaccurate or incomplete information, and data can follow patients as they move between providers. This structured data could be used as the basis for a visual longitudinal record (Figure 2(a)), providing an accessible summary of, say, disease severity against medication use through time. This can act as a visual aid during consultations, allowing patients and clinicians to jointly understand treatment response and make shared informed decisions, thereby providing a return on investment to the clinicians who enter structured data.

The NHS X Tech Plan for health and care states, ‘*we will know we have succeeded when clinicians find technology makes their working lives much easier [and] adding to clinical records, and looking things up from the whole of a patient’s record, become straightforward and intuitive*’.^11^ This is a laudable aim.

## A clearer picture of health through time: patient-generated data

Even if clinicians have a perfect view of what has happened until the last visit, we need patients to describe what has happened since then (Figure 2). It is well known that patients have difficulty recalling events from preceding months, and succinctly summarising day-to-day variations in symptoms (Figure 1(a)).

Integration of patient-generated data from consumer technologies into clinical care systems could be transformative by providing a more comprehensive picture of how patients live with their medical conditions, complementing provider-led capture of health-related data. Additionally, it is a way to capture and augment the patient voice and strengthen the patient–provider relationship. Collecting patient questionnaire data electronically from home prior to a visit could save time and resources in clinic, even more so if that was done longitudinally between clinics (Figures 1(b) and 2(b)). Passive monitoring using sensing technology is imagined to offer a viable, future alternative to long-term symptom tracking, though there are still hurdles to overcome before such ‘digital biomarkers’ are adopted in clinical care.^12^ Digital inclusion should be considered during development, implementation and evaluation to ensure that patients, even those with fewer digital skills, have the digital access, skills and confidence they need to contribute and benefit from digital health data.

Healthcare systems have been slow to formally integrate patient-generated data into EHRs. Efforts to date have predominantly been small-scale pilots in highly selected groups of participants (although non-integrated solutions are available and on the rise).^13^ This is due to the myriad of technical challenges, including data security and privacy, data standardisation, data analytics and visualisation, workflow integration and device interoperability, as well as patient and provider concerns.^14^ Nonetheless, it is achievable: in the UK, our Remote Monitoring of Rheumatoid Arthritis study uniquely integrated daily patient-reported symptoms from smartphones into the EHR, delivering proof that integrated systems are feasible and can transform consultations for clinician and patient benefit.^15^

## A strong data foundation for future outpatient care

Outpatient-based specialties share a common goal when collecting information: gather the necessary, accurate information to understand diagnoses, disease severity and treatment response, to inform decision making and effective communication. So, collect the right information, in the right way, and present it usefully. From clinicians, this means collecting coded, structured information while providing a ‘return on time investment’ by presenting a longitudinal view of disease severity and treatment response. From patients, a new infrastructure is needed to securely connect and present data collected between visits in the NHS. Together, this could underpin some of the national digital ambition, such as patient-initiated follow-up and ‘just in time’ interventions. It could also support a learning healthcare system that continually improves by collecting and processing data to inform better decision-making.

Realisation of this vision is within reach. It requires a change in how EHRs can best support collection of the right data from outpatients with useful real-time feedback. Uncoupling care organisations from the constrained functionality of their EHR providers would help. We are starting to learn how patient-generated data can be technically integrated into the NHS, how integrated patient data may lead to better health outcomes, the cost-effectiveness of remote patient monitoring, and what supporting materials are needed for both clinicians and patients. Despite its many challenges, we must strive to provide a solid data foundation for the inevitable changes in outpatient care in coming years to ensure we deliver safer, more efficient and more person-centred care.

## Declarations

### Guarantor

WGD.

## References

[1] Department of Health. Long term conditions compendium of information. See https://assets.publishing.service.gov.uk/government/uploads/system/uploads/attachment_data/file/216528/dh_134486.pdf (last checked 7 July 2021).

[2] NHS Digital. Hospital outpatient activity 2018–2019. See https://files.digital.nhs.uk/33/EF9007/hosp-epis-stat-outp-summ-rep-2018-19-rep.pdf (last checked 1 July 2021).

[3] Royal College of General Practioners. RCGP survey provides snapshot of how GP care is accessed in latest stages of pandemic. 2020. See www.rcgp.org.uk/about-us/news/2020/july/rcgp-survey-provides-snapshot-of-how-gp-care-is-accessed-in-latest-stages-of-pandemic.aspx (last checked 31 July 2021).

[4] National Health Service. The NHS long term plan. 2019. See www.longtermplan.nhs.uk/wp-content/uploads/2019/08/nhs-long-term-plan-version-1.2.pdf (last checked 31 July 2021).

[5] Department of Health and Social Care. Integration and innovation: working together to improve health and social care for all (HTML version). GOV.UK. 2021. See www.gov.uk/government/publications/working-together-to-improve-health-and-social-care-for-all/integration-and-innovation-working-together-to-improve-health-and-social-care-for-all-html-version (last checked 31 July 2021).

[6] Department of Health and Social Care. Data saves lives: reshaping health and social care with data (draft). GOV.UK. 2021. See www.gov.uk/government/publications/data-saves-lives-reshaping-health-and-social-care-with-data-draft/data-saves-lives-reshaping-health-and-social-care-with-data-draft (last checked 31 July 2021).

[7] National Institute of Health and Care Excellence. Shared decision making: Key theraputic topic [KTT23]. 2019. See www.nice.org.uk/advice/ktt23 (last checked 31 July 2021).

[8] Loudon IS . Historical importance of outpatients. Br Med J 1978; 1: 974–977. 346154 10.1136/bmj.1.6118.974 PMC1603853

[9] Person-Centred Care Team NHS England. Involving people in their own health and care. 2017. See www.england.nhs.uk/wp-content/uploads/2017/04/ppp-individual-involvement-equalties-analysis.pdf (last checked 31 July 2021).

[10] Warren LR Clarke J Arora S Darzi A . Improving data sharing between acute hospitals in England: an overview of health record system distribution and retrospective observational analysis of inter-hospital transitions of care. BMJ Open 2019; 9: e031637–e031637. 10.1136/bmjopen-2019-031637 PMC7008454 31806611

[11] NHS X. Tech Plan for health and care. See https://jointheconversation.scwcsu.nhs.uk/tech-plan (last checked 31 July 2021).

[12] Sim I . Mobile devices and health. N Engl J Med 2019; 381: 956–968. 31483966 10.1056/NEJMra1806949

[13] Gandrup J Ali SM McBeth J van der Veer SN Dixon WG . Remote symptom monitoring integrated into electronic health records: a systematic review. J Am Med Inform Assoc 2020; 27: 1752–1763. 32968785 10.1093/jamia/ocaa177 PMC7671621

[14] Office of the National Coordinator for Health Information Technology. Conceptualizing a data infrastructure for the capture, use, and sharing of patient-generated health data in care delivery and research through 2024. See www.healthit.gov/sites/default/files/onc_pghd_final_white_paper.pdf (last checked 31 July 2021).

[15] Austin L Sharp CA van der Veer SN , et al. Providing ‘the bigger picture’: benefits and feasibility of integrating remote monitoring from smartphones into the electronic health record: findings from the Remote Monitoring of Rheumatoid Arthritis (REMORA) study. Rheumatology 2020; 59: 367–378. 31335942 10.1093/rheumatology/kez207 PMC7223265

